# Role of the Bone Microenvironment in the Development of Painful Complications of Skeletal Metastases

**DOI:** 10.3390/cancers10050141

**Published:** 2018-05-10

**Authors:** Sun H. Park, Matthew R. Eber, D. Brooke Widner, Yusuke Shiozawa

**Affiliations:** Department of Cancer Biology and Comprehensive Cancer Center, Wake Forest School of Medicine, Winston-Salem, NC 27157, USA; shpark@wakehealth.edu (S.H.P.); meber@wakehealth.edu (M.R.E.); dwidner@wakehealth.edu (D.B.W.)

**Keywords:** cancer-induced bone pain, bone marrow microenvironment, osteoclasts, osteoblasts, macrophages, mast cells, stromal cells

## Abstract

Cancer-induced bone pain (CIBP) is the most common and painful complication in patients with bone metastases. It causes a significant reduction in patient quality of life. Available analgesic treatments for CIBP, such as opioids that target the central nervous system, come with severe side effects as well as the risk of abuse and addiction. Therefore, alternative treatments for CIBP are desperately needed. Although the exact mechanisms of CIBP have not been fully elucidated, recent studies using preclinical models have demonstrated the role of the bone marrow microenvironment (e.g., osteoclasts, osteoblasts, macrophages, mast cells, mesenchymal stem cells, and fibroblasts) in CIBP development. Several clinical trials have been performed based on these findings. CIBP is a complex and challenging condition that currently has no standard effective treatments other than opioids. Further studies are clearly warranted to better understand this painful condition and develop more effective and safer targeted therapies.

## 1. Introduction

Cancer-induced bone pain (CIBP) is the most common complication of bone metastases, and it significantly reduces the patient’s quality of life (QOL) [1]. CIBP poses a tremendous challenge to patients and their caregivers, in both managing it and identifying its underlying cause. In the quest for effective cancer therapy, maintaining QOL can be as crucial as treating the disease itself. Seventy-five percent of cancer patients experience pain throughout their disease [2]. Once cancer metastasizes to the bone, the first symptom is often acute bone pain. A full 80% of patients with bone metastases have CIBP [3].

CIBP remains a therapeutically challenging condition. It includes both spontaneous (ongoing) pain and breakthrough (movement-related) pain, which can present individually or in combination [4]. Unless each component of CIBP is appropriately treated, it cannot be managed. Analgesics for CIBP that target the central nervous system (e.g., opioids, nonsteroidal anti-inflammatory drugs or NSAIDs) are somewhat effective to reduce pain, but have severe side effects and are often extremely addictive [5,6,7]. External beam radiation, used for patients with bone metastases, is primarily palliative and only half of the patients receiving this therapy achieve partial or complete pain relief [4,8]. Alternatively, bisphosphonate and anti-receptor activator of nuclear factor κB ligand (RANKL) antibody treatments that decrease bone resorption in cancer patients with bone metastases [9,10], and radium-223 which forms complexes with hydroxylapatite in bone [11,12,13] can also reduce the onset of pain. However, even with these well-accepted clinical treatment modalities, 50% of patients with CIBP do not achieve controlled pain status [14].

CIBP is a very complex phenomenon that is uniquely distinct from other forms of chronic pain, such as inflammatory or neuropathic pain [15]. Bone is a richly innervated organ, and it has been suggested that bone metastatic cancer cells interact with sensory nerves in the bone microenvironment, resulting in CIBP development [15]. Mechanical stresses or mass effects from bone metastatic progression can directly induce CIBP, since sensory nerves that express mechanoreceptors reside throughout the interosseous membrane of long bones [16]. However, it has also been suggested that CIBP is not correlated with tumor type, location, number, or size of the metastases [17,18]. This may indicate that CIBP is developed not only during physical contact between bone metastatic cancer cells and sensory nerves, but also from signaling events initiated by cancer-derived factors [15,19,20,21]. Indeed, the acidic environment surrounding the tumor or cancer secreted growth factors, cytokines, or chemokines can all stimulate receptors on sensory nerves to induce CIBP [15,19,22,23].

The bone marrow provides a unique environment for both hematopoiesis and bone metastatic progression [24,25,26]. This microenvironment consists of several types of cells, including those that regulate bone remodeling, immune cells, stromal cells, and endothelial cells. It has been appreciated that the crosstalk between the bone microenvironment and bone metastatic cancer cells is crucial for bone metastatic progression [26,27,28]. However, little is known about how the molecular interactions between metastatic cancer and the bone marrow microenvironment affect CIBP.

Therefore, in this review, we provide a concise overview of the known roles of the bone marrow microenvironment in the development of CIBP and discuss the future directions of research on this topic.

## 2. The Roles of the Bone Marrow Microenvironment in the Development of Cancer-Induced Bone Pain

### 2.1. Osteoclasts

During bone metastatic progression, osteolytic cancer cells—originating from primary tumors from breast cancer, lung cancer, renal cancer, sarcomas, and multiple myeloma, etc.—stimulate osteoblasts to release RANKL. The osteoblast-derived RANKL binds to its receptor RANK expressed on osteoclasts. This interaction induces osteoclast maturation and increases osteolytic activity, resulting in enhanced bone resorption. Resorption of the bone matrix then causes the release of growth factors such as transforming growth factor beta (TGF-β) and insulin-like growth factor 1 (IGF-1), leading to further bone metastatic progression. The process whereby bone metastatic cancer cells establish osteolytic lesions to enhance bone metastatic progression is called the “vicious cycle” [27,29,30,31,32,33,34]. It has been suggested that enhanced osteoclast activity can also lead to CIBP. Consistent with this notion, increased mechanical and thermal hyperalgesia are observed in rodents inoculated with osteolytic osteosarcoma into the bone, compared to animals without tumors [35,36,37]. Administration of the decoy RANKL receptor osteoprotegerin (OPG) to osteosarcoma-bearing animals significantly decreased spontaneous pain behaviors [35,36]. However, OPG treatments did not affect tumor size.

During the bone resorption process, osteoclasts acidify (pH 4.0–4.5) the extracellular space by releasing protons and chloride ions through membrane transport (V-type H^+^ ATPase) [38]. The low pH condition sensitizes sensory nerves to mechanical, thermal, and chemical stimuli by activating acid sensing receptors such as the acid-sensing ion channels (ASICs) and transient receptor potential cation channel subfamily V member 1 (TRPV1) expressed on sensory nerves [39,40]. In addition, osteoclast activity indirectly causes TRPV1 upregulation in sensory nerves through the release of TGF-β and IGF-1 derived from the resorbed bone matrix. These factors increase the expression of their receptors (TGF-βRI and IGF-1R) on sensory nerves. In a rat CIBP model using mammary gland carcinoma, the upregulation of these growth factor receptors correlates with the upregulation and sensitization of TRPV1 in sensory nerves. The tumor-bearing animals in these studies had significantly increased mechanical and thermal sensitivity, and observed pain behaviors were reversed with treatments of TGF-βRI and IGF-1R antagonists [41,42].

Osteoclasts mediate bone resorption not only through the creation of acidic conditions, but also through adenosine triphosphate (ATP) production by mitochondrial cytochrome c oxidative activity [43]. Osteoclasts release accumulated intracellular ATP into the extracellular space, which can activate purinergic receptors such as P2X receptors, known as the ATP-gated ion channels [44,45]. It has been demonstrated that: (1) approximately 90% of the peripheral and central sensory neurons express P2X receptors; and (2) the subtypes of P2X receptors including P2X4, P2X3, and P2X7 regulate the development of neuropathic and inflammatory pain [46,47]. Specifically, P2X3 and P2X2/3 expressed on the terminal end of primary afferent neurons innervating bone have been shown to be involved in CIBP development [48,49,50,51,52]. For instance, in C3H/HeJ mice receiving intratibial inoculations of osteolytic fibrosarcoma, increased expression of P2X3 receptor in the peripheral nociceptive fibers is observed, and peri-tumoral injections of receptor antagonist naloxone-methiodide (A-317491) inhibited cancer-mediated thermal hyperalgesia [52]. Moreover, growing tumor cells also generate ATP [53,54], and ATP itself stimulates osteoclast activities through the P2X3 receptor [55,56]. Therefore, ATP can induce CIBP by directly interacting with P2X receptors on sensory nerves or indirectly through enhanced osteoclastic activity. Although further studies are warranted, pharmacologically targeting the ATP/P2X receptors axis may prove to be a potential therapeutic strategy for CIBP.

### 2.2. Osteoblasts

Unlike osteolytic bone metastatic progression, the mechanisms driving osteoblastic bone metastatic progression are largely unknown. Prostate cancer is one of the primary cancer types that gives rise to developing osteoblastic lesions. It has been demonstrated that prostate cancer cells facilitate new bone formation by secreting parathyroid hormone-related protein (PTHrP) [57], urokinase-type plasminogen activator (uPA) [58], or prostate-specific antigen (PSA) [59]. Endothelin-1 (ET-1), a vasoconstrictor, is also known as an osteoblast inducing factor. It stimulates mitogenesis of osteoblasts when it binds to endothelin A receptor (ETAR) and endothelin B receptor (ETBR) expressed by osteoblasts [60,61]. When the osteolytic human breast cancer cell line ZR-75-1 was made to overexpress ET-1, osteoblastic metastases along with new bone formation were detected after inoculation into murine bone, and treatments with the ETAR selective antagonist (ABT-627) attenuated these osteoblastic lesions [62]. The ET-1/ETAR interaction also plays important roles in CIBP development [20,63]. In fact, the administration of ABT-627 into ET-1 expressing osteosarcoma-bearing mice attenuates CIBP behaviors, but the ETBR selective antagonist, A-192621, does not reduce CIBP behavior [20]. Consistently, phase II and III clinical trials in patients with hormone refractory prostate cancer showed that ABT-627 delayed the time to bone alkaline phosphatase progression and reduced bone pain, compared to those with placebo [64,65]. Additionally, local injection of ET-1 into the peri-tumoral area of a fibrosarcoma bone cancer murine model increased pain behaviors, and the ETAR antagonist BQ-123 inhibited these behaviors [66]. A meta-analysis of 9 clinical studies examining the effects of ETAR antagonists on castration-resistant prostate cancer patients, consistently showed that the ETAR antagonist Atrasentan reduces the relative risk of bone pain [67].

Newly formed bone or woven bone that is mediated by bone metastatic cancer cells is weaker than normal bone. While normal bone has a regular parallel alignment of collagen sheets and is mechanically strong, newly formed immature bone is made of a smaller number of randomly oriented collagen fibers and is mechanically weak, although it forms quickly [68,69]. Therefore, the weakening and instability of new tumor-bearing bone, easily leads to bone damage and CIBP. A canine prostate cancer cell line ACE-1 establishes osteoblastic bone metastatic lesions and develops spontaneous and mechanical pain behaviors in vivo [70]. Since the studies to reveal the impact of tumor-bearing newly formed bone on CIBP are underdeveloped, the use of this model can be helpful to further determine the detailed molecular mechanisms.

### 2.3. Immune Cells

Inflammation is also involved in the pathobiology of CIBP via several immune cell types (e.g., macrophages and mast cells) [71]. Macrophages are known to contribute to the tumor microenvironment, and they release inflammatory factors upon interacting with tumor cells. These inflammatory factors can induce both disease progression and cancer-related pain [72,73,74]. Some types of cancer cells that metastasize to the bone (e.g., breast cancer, prostate cancer) highly express and secrete the neurotrophins nerve growth factor (NGF) and brain-derived neurotrophic factor (BDNF) [75,76]. Macrophages express the functional receptors for NGF [p75 neurotrophin receptor (p75NTR), tropomyosin receptor kinase A (TrkA)] and BDNF [tropomyosin receptor kinase B (TrkB)] [77]. NGF and BDNF derived from bone metastatic cancer cells activate macrophages to release pro-inflammatory cytokines [tumor necrosis factor-alpha (TNF-α), interleukin (IL)-6, IL-1β] and inflammatory regulators [NGF, Prostaglandin E_2_ (PGE_2_)] that sensitize nociceptors [78,79]. In addition, as their names indicate, NGF and BDNF also directly regulate the survival, development, and function of neurons [80].

IL-1β can stimulate expression of cyclooxygenase 2 (Cox-2), and Cox-2 facilitates synthesis of prostaglandins in macrophages [81]. Prostaglandins can sensitize or activate the sensory nerves by binding to prostanoid receptors, resulting in CIBP [82]. The administration of a Cox-2 inhibitor into an osteosarcoma CIBP model inhibits pain behaviors as well as bone destruction without affecting tumor burden [82,83,84,85,86]. Since most NSAIDs inhibit both Cox-1 and Cox-2, which are required for PGE_2_ synthesis [87], and the major adverse events mediated by NSAIDs are thought to be Cox-1 dependent [88,89], blocking Cox-2 release from macrophages may be a better option for CIBP treatment than NSAIDs. However, a randomized, Phase III trial between a selective Cox-2 inhibitor (rofecoxib) and a non-selective NSAID (naproxen) in patients with rheumatoid arthritis revealed that chronic administration of rofecoxib causes more myocardial infarction than naproxen, while there is no significant difference in treatment efficacy between the two [90]. A more recent pre-clinical study using a lung carcinoma CIBP mouse model demonstrated that when microsomal PGE synthase-1 (mPGES-1), another prostaglandin synthesizing enzyme, is deleted, the onset of pain behaviors mediated by the growth of lung carcinoma in the tibia is delayed [91]. These findings suggest that targeting Cox-2 expression and PGE_2_ synthesis in macrophages can be potential therapeutic strategies for CIBP. However, further studies are needed to elucidate the pathophysiology of adverse events before moving into the clinic.

Pre-clinical murine CIBP studies of osteosarcoma, breast cancer, and prostate cancer have demonstrated that blockage of NGF significantly attenuates pain behaviors and bone destruction mediated by bone tumors [92,93,94]. Additionally, an anti-NGF monoclonal antibody tanezumab was clinically tested in patients with osteoarthritis and diabetic peripheral neuropathy, and overall significant pain relief is observed in patients treated with tanezumab compared to those treated with placebo [95,96,97,98]. Although larger clinical trials will be needed, a recent clinical study in patients with bone metastatic prostate cancer, breast cancer, renal cell carcinoma, or multiple myeloma (placebo *n* = 30, tanezumab *n* = 29) demonstrated greater efficacy in pain relief in patients treated with tanezumab than placebo treated patients [99].

Protease-activated receptors (PAR-2), a sub-family of G protein-coupled receptors that are highly expressed on sensory nerves, are known to be involved in the development of inflammatory and neuropathic pain in rodent models [100,101,102]. PAR-2 is mainly activated by mast cell tryptase and trypsin [103,104,105]. Mast cells are located near sensory neurons; contain cytoplasmic granules that store inflammatory mediators; and their release of pain transmitters causes pain [106,107,108]. When the conditioned medium obtained from human squamous cell carcinoma is injected into the mouse hind paw, the skin mast cells are activated and increased pain behaviors are observed [109]. However, this cancer-associated mechanical allodynia is reversed with treatments of the tryptase inhibitor APC-366 or soybean trypsin inhibitor (SBTI) [109]. Activation of PAR-2 has been shown to increase levels of neuropeptides such as calcitonin gene-related peptide (CGRP) and substance P (SP). Sensory nerve sprouting from CGRP expressing neurons is known to be associated with skeletal pain behaviors [110,111,112,113], and levels of plasma CGRP directly correlate with the pain intensity experienced in several pain related conditions [114,115]. It has been demonstrated that bone tumor enhances the PAR-2 expression in sensory nerves [116]. In addition, recent studies have revealed that tumor-infiltrating mast cells in bone metastatic tumors of gastric cancer promote bone metastatic growth, osteolytic lesions, and CIBP by stimulating angiogenesis [117,118].

### 2.4. Stromal Cells

Bone is a hypoxic tissue (pO2: 8.1–26.7 mmHg) [119,120], and this hypoxic environment is crucial for controlling angiogenesis [121], bone repair [122], osteoblastogenesis [123], osteoclastogenesis [124], and hematopoiesis [125]. Moreover, under hypoxia, tumor cells generate large amounts of lactate through elevated levels of aerobic glycolysis, leading to a lowering of intracellular pH (pH 6.8). This is known as the Warburg effect [53,126]. To prevent cell death mediated by this intracellular acidification, tumor cells actively pump out the protons and lactate to the extracellular space. This extracellular acidic environment surrounding tumor cells can stimulate the cells of stromal origin in the marrow, such as mesenchymal stem cells (MSCs) and fibroblasts [127]. Bone marrow MSCs and fibroblasts are known to express high levels of acid sensing receptors [acid-sensing ion channel 3 (ASIC3), ASIC4, G protein-coupled receptor 4 (GPR4), and GPR65] [128], and become activated by the acidic environment created by bone metastatic tumor cells. This interaction leads to expression and secretion of inflammatory cytokines [IL-6, IL-8, IL-15, chemokine (C-C motif) ligand 5 (CCL5), IL-1ß] as well as nociceptive mediators such as NGF and BDNF [128]. Therefore, it has been suggested that bone metastatic tumor cells induce CIBP by interacting with bone marrow stromal cells.

## 3. Discussion

**Figure 1:**
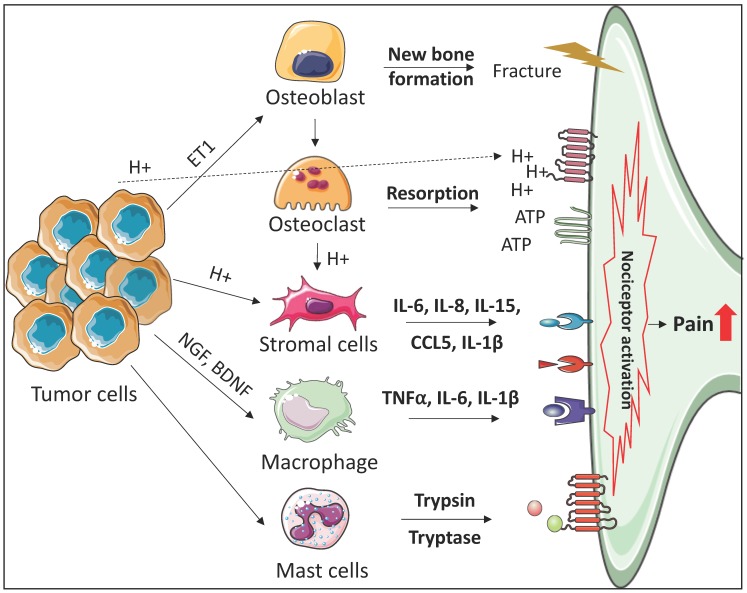
Mechanisms of bone microenvironment involvement in cancer-induced bone pain. Bone-disseminated tumor cells release factors (e.g., ET1) to stimulate the proliferation of osteoblasts (e.g., endothelin A/B receptors), resulting in new bone formation, which is structurally weak and prone to fracture. Active osteoblasts release RANKL to promote osteoclast activity, resulting in increased bone resorption which also weakens bone. During bone resorption, nociceptors become sensitized and activated through osteoclast mediated acidification and ATP accumulation, which activates the acid sensing TRPV1 and ASICS receptors, or the ATP-gated P2X receptors expressed on sensory neurons, respectively. Tumor cell derived H+ directly induces nociception via activation of the acid sensing receptors expressed on the sensory neurons. Stromal cells (e.g., fibroblasts and mesenchymal stem cells) also express acid sensing receptors, and acidification of the bone marrow space stimulates release of stromal cell derived pro-inflammatory cytokines (IL-6, IL-8, IL-15, CCL5, IL-1β) and nociceptive mediators (NGF and BDNF). Tumor cells also express NGF and BDNF, which activate macrophages to release pro-inflammatory cytokines (TNF-α, IL-6, IL-1β) and inflammatory regulators (NGF and Prostaglandins) which directly induce pain via binding to their receptors on sensory neurons. Finally, tumor cells interact with peri-neural and tumor-infiltrated mast cells, releasing mast cell derived proteases (trypsin and tryptase) which activate sensory neurons by binding to PAR-2 receptor, resulting in pain and upregulation of pain-related neuropeptides (CGRP and SP).

Despite the improvement of current cytotoxic treatments, these treatments may not provide survival benefits to all advanced cancer patients. However, most of these patients suffer from symptoms that negatively impact their QOL, such as CIBP. CIBP is a very complex symptom since bone metastatic cancer, sensory nerves, and the bone microenvironment interact together to cause such a painful condition. Therefore, revealing the detailed mechanisms whereby the components that are responsible for bone metastatic progression are involved in the CIBP development will be very important in furthering understanding of this painful symptom and possibly for the development of effective therapies. In this review, we discussed the roles of the cells controlling bone remodeling, immune cells, and stromal cells in the development of CIBP (Figure 1). However, these findings are based on limited evidence. Further studies are therefore clearly needed in this area.

It has been suggested that higher patient QOL may indicate a survival benefit [129]. Along with this notion, recent research suggests that CIBP may be both a reason for decreased QOL and an indicator of survival [130,131,132,133]. For instance, the ALSYMPCA trial [134], which investigated the role of radium-223 in patients with prostate cancer and bone metastases, demonstrated that decreased pain levels correlated with increased overall survival. Additionally, the importance of nerves for cancer development has been appreciated [135]. Recent studies revealed that the sympathetic nervous system regulates the metastatic process of prostate cancer to bone [136], and that denervation can suppress tumorigenesis and metastasis [136,137,138,139]. However, it remains unclear whether sensory nerves that innervate bone, which are responsible for bone pain, also promote metastatic progression to bone. Therefore, revealing the mechanisms of CIBP may provide a strong foundation for much-needed treatments for bone metastases. Stopping pain signals may be useful to improve both morbidity and mortality.

## 4. Conclusions

The current first-line treatment for CIBP is still opioids. Opioids are somewhat effective, but can have serious side effects, and abuse and addiction of these analgesics are a growing concern. Therefore, more effective and safer alternative treatment options for CIBP are urgently needed. Unfortunately, there is currently no better treatment for CIBP than opioids, when administered alone. However, several lines of evidence suggest that the combination of non-opioid analgesics with opioids provides synergistic or additive analgesic effects that can lead to decreased opioid dose. In the case of CIBP, the combination treatment of PAR-2 antagonists with morphine can allow the use of significantly lower doses of morphine (1 mg/kg) while maintaining the same levels of analgesia as single high doses of morphine (3 or 10 mg/kg) in a CIBP rat model [140]. Although it might be difficult to immediately replace opioids with other treatment modalities, we still need to continue efforts to reduce opioid use by discovering potential therapeutic targets for CIBP within the bone marrow microenvironment.

## References

[1] Tsuzuki S., Park S.H., Eber M.R., Peters C.M., Shiozawa Y. (2016). Skeletal complications in cancer patients with bone metastases. Int. J. Urol..

[2] Meuser T., Pietruck C., Radbruch L., Stute P., Lehmann K.A., Grond S. (2001). Symptoms during cancer pain treatment following who-guidelines: A longitudinal follow-up study of symptom prevalence, severity and etiology. Pain.

[3] Berruti A., Dogliotti L., Bitossi R., Fasolis G., Gorzegno G., Bellina M., Torta M., Porpiglia F., Fontana D., Angeli A. (2000). Incidence of skeletal complications in patients with bone metastatic prostate cancer and hormone refractory disease: Predictive role of bone resorption and formation markers evaluated at baseline. J. Urol..

[4] Laird B.J., Walley J., Murray G.D., Clausen E., Colvin L.A., Fallon M.T. (2011). Characterization of cancer-induced bone pain: An exploratory study. Support Care Cancer.

[5] Benyamin R., Trescot A.M., Datta S., Buenaventura R., Adlaka R., Sehgal N., Glaser S.E., Vallejo R. (2008). Opioid complications and side effects. Pain Phys..

[6] Pergolizzi J., Boger R.H., Budd K., Dahan A., Erdine S., Hans G., Kress H.G., Langford R., Likar R., Raffa R.B. (2008). Opioids and the management of chronic severe pain in the elderly: Consensus statement of an international expert panel with focus on the six clinically most often used world health organization step III opioids (buprenorphine, fentanyl, hydromorphone, methadone, morphine, oxycodone). Pain Pract..

[7] Mercadante S. (2001). The use of anti-inflammatory drugs in cancer pain. Cancer Treat. Rev..

[8] De Felice F., Piccioli A., Musio D., Tombolini V. (2017). The role of radiation therapy in bone metastases management. Oncotarget.

[9] Stopeck A.T., Lipton A., Body J.J., Steger G.G., Tonkin K., de Boer R.H., Lichinitser M., Fujiwara Y., Yardley D.A., Viniegra M. (2010). Denosumab compared with zoledronic acid for the treatment of bone metastases in patients with advanced breast cancer: A randomized, double-blind study. J. Clin. Oncol..

[10] Fizazi K., Carducci M., Smith M., Damiao R., Brown J., Karsh L., Milecki P., Shore N., Rader M., Wang H. (2011). Denosumab versus zoledronic acid for treatment of bone metastases in men with castration-resistant prostate cancer: A randomised, double-blind study. Lancet.

[11] Badrising S.K., van der Noort V., Hamberg P., Coenen J.L., Aarts M.J., van Oort I.M., van den Eertwegh A.J., Los M., van den Berg H.P., Gelderblom H. (2016). Enzalutamide as a fourth- or fifth-line treatment option for metastatic castration-resistant prostate cancer. Oncology.

[12] Vignani F., Bertaglia V., Buttigliero C., Tucci M., Scagliotti G.V., Di Maio M. (2016). Skeletal metastases and impact of anticancer and bone-targeted agents in patients with castration-resistant prostate cancer. Cancer Treat. Rev..

[13] Abou D.S., Ulmert D., Doucet M., Hobbs R.F., Riddle R.C., Thorek D.L. (2016). Whole-body and microenvironmental localization of radium-223 in naive and mouse models of prostate cancer metastasis. J. Natl. Cancer Inst..

[14] Delaney A., Fleetwood-Walker S.M., Colvin L.A., Fallon M. (2008). Translational medicine: Cancer pain mechanisms and management. Br. J. Anaesth..

[15] Lozano-Ondoua A.N., Symons-Liguori A.M., Vanderah T.W. (2013). Cancer-induced bone pain: Mechanisms and models. Neurosci. Lett..

[16] Rowe M.J., Tracey D.J., Mahns D.A., Sahai V., Ivanusic J.J. (2005). Mechanosensory perception: Are there contributions from bone-associated receptors?. Clin. Exp. Pharmacol. Physiol..

[17] Nieder C., Pawinski A., Dalhaug A. (2013). Continuous controversy about radiation oncologists’ choice of treatment regimens for bone metastases: Should we blame doctors, cancer-related features, or design of previous clinical studies?. Radiat. Oncol..

[18] Carrafiello G., Lagana D., Pellegrino C., Mangini M., Fontana F., Piacentino F., Recaldini C., Rovera F., Dionigi G., Boni L. (2008). Ablation of painful metastatic bone tumors: A systematic review. Int. J. Surg..

[19] Mantyh P.W., Clohisy D.R., Koltzenburg M., Hunt S.P. (2002). Molecular mechanisms of cancer pain. Nat. Rev. Cancer.

[20] Peters C.M., Lindsay T.H., Pomonis J.D., Luger N.M., Ghilardi J.R., Sevcik M.A., Mantyh P.W. (2004). Endothelin and the tumorigenic component of bone cancer pain. Neuroscience.

[21] Sevcik M.A., Ghilardi J.R., Peters C.M., Lindsay T.H., Halvorson K.G., Jonas B.M., Kubota K., Kuskowski M.A., Boustany L., Shelton D.L. (2005). Anti-NGF therapy profoundly reduces bone cancer pain and the accompanying increase in markers of peripheral and central sensitization. Pain.

[22] Ghilardi J.R., Rohrich H., Lindsay T.H., Sevcik M.A., Schwei M.J., Kubota K., Halvorson K.G., Poblete J., Chaplan S.R., Dubin A.E. (2005). Selective blockade of the capsaicin receptor TRPV1 attenuates bone cancer pain. J. Neurosci..

[23] Yoneda T., Hata K., Nakanishi M., Nagae M., Nagayama T., Wakabayashi H., Nishisho T., Sakurai T., Hiraga T. (2011). Involvement of acidic microenvironment in the pathophysiology of cancer-associated bone pain. Bone.

[24] Wilson A., Trumpp A. (2006). Bone-marrow haematopoietic-stem-cell niches. Nat. Rev. Immunol..

[25] Yin T., Li L. (2006). The stem cell niches in bone. J. Clin. Investig..

[26] Shiozawa Y., Eber M.R., Berry J.E., Taichman R.S. (2015). Bone marrow as a metastatic niche for disseminated tumor cells from solid tumors. Bonekey Rep..

[27] Zheng Y., Zhou H., Dunstan C.R., Sutherland R.L., Seibel M.J. (2013). The role of the bone microenvironment in skeletal metastasis. J. Bone Oncol..

[28] Buenrostro D., Park S.I., Sterling J.A. (2014). Dissecting the role of bone marrow stromal cells on bone metastases. BioMed Res. Int..

[29] Chirgwin J.M., Guise T.A. (2000). Molecular mechanisms of tumor-bone interactions in osteolytic metastases. Crit. Rev. Eukaryot. Gene Expr..

[30] Guise T.A., Kozlow W.M., Heras-Herzig A., Padalecki S.S., Yin J.J., Chirgwin J.M. (2005). Molecular mechanisms of breast cancer metastases to bone. Clin. Breast Cancer.

[31] Guise T.A., Mohammad K.S., Clines G., Stebbins E.G., Wong D.H., Higgins L.S., Vessella R., Corey E., Padalecki S., Suva L. (2006). Basic mechanisms responsible for osteolytic and osteoblastic bone metastases. Clin. Cancer Res..

[32] Kakonen S.M., Mundy G.R. (2003). Mechanisms of osteolytic bone metastases in breast carcinoma. Cancer.

[33] Kingsley L.A., Fournier P.G., Chirgwin J.M., Guise T.A. (2007). Molecular biology of bone metastasis. Mol. Cancer Ther..

[34] Mundy G.R. (1997). Mechanisms of bone metastasis. Cancer.

[35] Honore P., Luger N.M., Sabino M.A., Schwei M.J., Rogers S.D., Mach D.B., O’Keefe P F., Ramnaraine M.L., Clohisy D.R., Mantyh P.W. (2000). Osteoprotegerin blocks bone cancer-induced skeletal destruction, skeletal pain and pain-related neurochemical reorganization of the spinal cord. Nat. Med..

[36] Luger N.M., Honore P., Sabino M.A., Schwei M.J., Rogers S.D., Mach D.B., Clohisy D.R., Mantyh P.W. (2001). Osteoprotegerin diminishes advanced bone cancer pain. Cancer Res..

[37] Schwei M.J., Honore P., Rogers S.D., Salak-Johnson J.L., Finke M.P., Ramnaraine M.L., Clohisy D.R., Mantyh P.W. (1999). Neurochemical and cellular reorganization of the spinal cord in a murine model of bone cancer pain. J. Neurosci..

[38] Qin A., Cheng T.S., Pavlos N.J., Lin Z., Dai K.R., Zheng M.H. (2012). V-atpases in osteoclasts: Structure, function and potential inhibitors of bone resorption. Int. J. Biochem. Cell Biol..

[39] Lingueglia E. (2007). Acid-sensing ion channels in sensory perception. J. Biol. Chem..

[40] Holzer P. (2009). Acid-sensitive ion channels and receptors. Handb. Exp. Pharmacol..

[41] Li Y., Cai J., Han Y., Xiao X., Meng X.L., Su L., Liu F.Y., Xing G.G., Wan Y. (2014). Enhanced function of TRPV1 via up-regulation by insulin-like growth factor-1 in a rat model of bone cancer pain. Eur. J. Pain.

[42] Xu Q., Zhang X.M., Duan K.Z., Gu X.Y., Han M., Liu B.L., Zhao Z.Q., Zhang Y.Q. (2013). Peripheral TGF-beta1 signaling is a critical event in bone cancer-induced hyperalgesia in rodents. J. Neurosci..

[43] Kadenbach B., Huttemann M., Arnold S., Lee I., Bender E. (2000). Mitochondrial energy metabolism is regulated via nuclear-coded subunits of cytochrome C oxidase. Free Radic. Biol. Med..

[44] Brandao-Burch A., Key M.L., Patel J.J., Arnett T.R., Orriss I.R. (2012). The P2X7 receptor is an important regulator of extracellular ATP levels. Front. Endocrinol. (Lausanne).

[45] Reyes J.P., Sims S.M., Dixon S.J. (2011). P2 receptor expression, signaling and function in osteoclasts. Front. Biosci. (Schol. Ed.).

[46] North R.A. (2004). P2X3 receptors and peripheral pain mechanisms. J. Physiol..

[47] Wirkner K., Sperlagh B., Illes P. (2007). P2X3 receptor involvement in pain states. Mol. Neurobiol..

[48] Kaan T.K., Yip P.K., Patel S., Davies M., Marchand F., Cockayne D.A., Nunn P.A., Dickenson A.H., Ford A.P., Zhong Y. (2010). Systemic blockade of P2X3 and P2X2/3 receptors attenuates bone cancer pain behaviour in rats. Brain.

[49] Burnstock G. (2000). P2X receptors in sensory neurones. Br. J. Anaesth..

[50] Hansen R.R., Nasser A., Falk S., Baldvinsson S.B., Ohlsson P.H., Bahl J.M., Jarvis M.F., Ding M., Heegaard A.M. (2012). Chronic administration of the selective P2X3, P2X2/3 receptor antagonist, A-317491, transiently attenuates cancer-induced bone pain in mice. Eur. J. Pharmacol..

[51] Wu J.X., Xu M.Y., Miao X.R., Lu Z.J., Yuan X.M., Li X.Q., Yu W.F. (2012). Functional up-regulation of p2X3 receptors in dorsal root ganglion in a rat model of bone cancer pain. Eur. J. Pain.

[52] Gonzalez-Rodriguez S., Pevida M., Roques B.P., Fournie-Zaluski M.C., Hidalgo A., Menendez L., Baamonde A. (2009). Involvement of enkephalins in the inhibition of osteosarcoma-induced thermal hyperalgesia evoked by the blockade of peripheral P2X3 receptors. Neurosci. Lett..

[53] Epstein T., Gatenby R.A., Brown J.S. (2017). The warburg effect as an adaptation of cancer cells to rapid fluctuations in energy demand. PLoS ONE.

[54] Grygorczyk R., Furuya K., Sokabe M. (2013). Imaging and characterization of stretch-induced ATP release from alveolar a549 cells. J. Physiol..

[55] Hoebertz A., Townsend-Nicholson A., Glass R., Burnstock G., Arnett T.R. (2000). Expression of P2 receptors in bone and cultured bone cells. Bone.

[56] Morrison M.S., Turin L., King B.F., Burnstock G., Arnett T.R. (1998). ATP is a potent stimulator of the activation and formation of rodent osteoclasts. J. Physiol..

[57] Liao J., Li X., Koh A.J., Berry J.E., Thudi N., Rosol T.J., Pienta K.J., McCauley L.K. (2008). Tumor expressed PTHRP facilitates prostate cancer-induced osteoblastic lesions. Int. J. Cancer.

[58] Achbarou A., Kaiser S., Tremblay G., Ste-Marie L.G., Brodt P., Goltzman D., Rabbani S.A. (1994). Urokinase overproduction results in increased skeletal metastasis by prostate cancer cells in vivo. Cancer Res..

[59] Killian C.S., Corral D.A., Kawinski E., Constantine R.I. (1993). Mitogenic response of osteoblast cells to prostate-specific antigen suggests an activation of latent TGF-beta and a proteolytic modulation of cell adhesion receptors. Biochem. Biophys. Res. Commun..

[60] Kitano Y., Kurihara H., Kurihara Y., Maemura K., Ryo Y., Yazaki Y., Harii K. (1998). Gene expression of bone matrix proteins and endothelin receptors in endothelin-1-deficient mice revealed by in situ hybridization. J. Bone Miner. Res..

[61] Kasperk C.H., Borcsok I., Schairer H.U., Schneider U., Nawroth P.P., Niethard F.U., Ziegler R. (1997). Endothelin-1 is a potent regulator of human bone cell metabolism in vitro. Calcif. Tissue Int..

[62] Yin J.J., Mohammad K.S., Kakonen S.M., Harris S., Wu-Wong J.R., Wessale J.L., Padley R.J., Garrett I.R., Chirgwin J.M., Guise T.A. (2003). A causal role for endothelin-1 in the pathogenesis of osteoblastic bone metastases. Proc. Natl. Acad. Sci. USA.

[63] Pomonis J.D., Rogers S.D., Peters C.M., Ghilardi J.R., Mantyh P.W. (2001). Expression and localization of endothelin receptors: Implications for the involvement of peripheral GLIA in nociception. J. Neurosci..

[64] Nelson J.B., Nabulsi A.A., Vogelzang N.J., Breul J., Zonnenberg B.A., Daliani D.D., Schulman C.C., Carducci M.A. (2003). Suppression of prostate cancer induced bone remodeling by the endothelin receptor a antagonist atrasentan. J. Urol..

[65] Carducci M.A., Saad F., Abrahamsson P.A., Dearnaley D.P., Schulman C.C., North S.A., Sleep D.J., Isaacson J.D., Nelson J.B., Atrasentan Phase I.I.I.S.G.I. (2007). A phase 3 randomized controlled trial of the efficacy and safety of Atrasentan in men with metastatic hormone-refractory prostate cancer. Cancer.

[66] Wacnik P.W., Eikmeier L.J., Ruggles T.R., Ramnaraine M.L., Walcheck B.K., Beitz A.J., Wilcox G.L. (2001). Functional interactions between tumor and peripheral nerve: Morphology, algogen identification, and behavioral characterization of a new murine model of cancer pain. J. Neurosci..

[67] Qiao L., Liang Y., Li N., Hu X., Luo D., Gu J., Lu Y., Zheng Q. (2015). Endothelin-A receptor antagonists in prostate cancer treatment-a meta-analysis. Int. J. Clin. Exp. Med..

[68] Weiner S., Wagner H.D. (1998). The material bone: Structure-mechanical function relations. Annu. Rev. Mater. Sci..

[69] Prondvai E., Stein K.H.W., de Ricqlès A., Cubo J. (2014). Development-based revision of bone tissue classification: The importance of semantics for science. Biol. J. Linn. Soc..

[70] Halvorson K.G., Sevcik M.A., Ghilardi J.R., Rosol T.J., Mantyh P.W. (2006). Similarities and differences in tumor growth, skeletal remodeling and pain in an osteolytic and osteoblastic model of bone cancer. Clin. J. Pain.

[71] Raoof R., Willemen H., Eijkelkamp N. (2018). Divergent roles of immune cells and their mediators in pain. Rheumatology (Oxford).

[72] Hiraoka K., Zenmyo M., Watari K., Iguchi H., Fotovati A., Kimura Y.N., Hosoi F., Shoda T., Nagata K., Osada H. (2008). Inhibition of bone and muscle metastases of lung cancer cells by a decrease in the number of monocytes/macrophages. Cancer Sci..

[73] Sawa-Wejksza K., Kandefer-Szerszen M. (2018). Tumor-associated macrophages as target for antitumor therapy. Arch. Immunol. Ther. Exp..

[74] Zelenka M., Schafers M., Sommer C. (2005). Intraneural injection of interleukin-1beta and tumor necrosis factor-alpha into rat sciatic nerve at physiological doses induces signs of neuropathic pain. Pain.

[75] Adriaenssens E., Vanhecke E., Saule P., Mougel A., Page A., Romon R., Nurcombe V., Le Bourhis X., Hondermarck H. (2008). Nerve growth factor is a potential therapeutic target in breast cancer. Cancer Res..

[76] Hondermarck H. (2012). Neurotrophins and their receptors in breast cancer. Cytokine Growth Factor Rev..

[77] Williams K.S., Killebrew D.A., Clary G.P., Seawell J.A., Meeker R.B. (2015). Differential regulation of macrophage phenotype by mature and pro-nerve growth factor. J. Neuroimmunol..

[78] Zhang X.C., Kainz V., Burstein R., Levy D. (2011). Tumor necrosis factor-alpha induces sensitization of meningeal nociceptors mediated via local COX and P38 map kinase actions. Pain.

[79] Binshtok A.M., Wang H., Zimmermann K., Amaya F., Vardeh D., Shi L., Brenner G.J., Ji R.R., Bean B.P., Woolf C.J. (2008). Nociceptors are interleukin-1beta sensors. J. Neurosci..

[80] Huang E.J., Reichardt L.F. (2001). Neurotrophins: Roles in neuronal development and function. Annu. Rev. Neurosci..

[81] Barrios-Rodiles M., Chadee K. (1998). Novel regulation of cyclooxygenase-2 expression and prostaglandin e2 production by IFN-gamma in human macrophages. J. Immunol..

[82] Sabino M.C., Ghilardi J.R., Feia K.J., Jongen J.L., Keyser C.P., Luger N.M., Mach D.B., Peters C.M., Rogers S.D., Schwei M.J. (2002). The involvement of prostaglandins in tumorigenesis, tumor-induced osteolysis and bone cancer pain. J. Musculoskelet. Neuronal Interact..

[83] Fox A., Medhurst S., Courade J.P., Glatt M., Dawson J., Urban L., Bevan S., Gonzalez I. (2004). Anti-hyperalgesic activity of the COX-2 inhibitor lumiracoxib in a model of bone cancer pain in the rat. Pain.

[84] Sabino M.A., Ghilardi J.R., Jongen J.L., Keyser C.P., Luger N.M., Mach D.B., Peters C.M., Rogers S.D., Schwei M.J., de Felipe C. (2002). Simultaneous reduction in cancer pain, bone destruction, and tumor growth by selective inhibition of cyclooxygenase-2. Cancer Res..

[85] Bottner F., Roedl R., Wortler K., Grethen C., Winkelmann W., Lindner N. (2001). Cyclooxygenase-2 inhibitor for pain management in osteoid osteoma. Clin. Orthop. Relat. Res..

[86] Carpintero-Benitez P., Aguirre M.A., Serrano J.A., Lluch M. (2004). Effect of rofecoxib on pain caused by osteoid osteoma. Orthopedics.

[87] Vane J.R. (1996). Introduction: Mechanism of action of NSAIDs. Br. J. Rheumatol..

[88] Laneuville O., Breuer D.K., Dewitt D.L., Hla T., Funk C.D., Smith W.L. (1994). Differential inhibition of human prostaglandin endoperoxide h synthases-1 and -2 by nonsteroidal anti-inflammatory drugs. J. Pharmacol. Exp. Ther..

[89] Antman E.M., Bennett J.S., Daugherty A., Furberg C., Roberts H., Taubert K.A. (2007). Use of nonsteroidal antiinflammatory drugs. Update Clin. Sci. Statement Am. Heart Assoc..

[90] Bombardier C., Laine L., Reicin A., Shapiro D., Burgos-Vargas R., Davis B., Day R., Ferraz M.B., Hawkey C.J., Hochberg M.C. (2000). Comparison of upper gastrointestinal toxicity of rofecoxib and naproxen in patients with rheumatoid arthritis. Vigor study group. N. Engl. J. Med..

[91] Isono M., Suzuki T., Hosono K., Hayashi I., Sakagami H., Uematsu S., Akira S., DeClerck Y.A., Okamoto H., Majima M. (2011). Microsomal prostaglandin e synthase-1 enhances bone cancer growth and bone cancer-related pain behaviors in mice. Life Sci..

[92] McCaffrey G., Thompson M.L., Majuta L., Fealk M.N., Chartier S., Longo G., Mantyh P.W. (2014). Ngf blockade at early times during bone cancer development attenuates bone destruction and increases limb use. Cancer Res..

[93] Halvorson K.G., Kubota K., Sevcik M.A., Lindsay T.H., Sotillo J.E., Ghilardi J.R., Rosol T.J., Boustany L., Shelton D.L., Mantyh P.W. (2005). A blocking antibody to nerve growth factor attenuates skeletal pain induced by prostate tumor cells growing in bone. Cancer Res..

[94] Bloom A.P., Jimenez-Andrade J.M., Taylor R.N., Castaneda-Corral G., Kaczmarska M.J., Freeman K.T., Coughlin K.A., Ghilardi J.R., Kuskowski M.A., Mantyh P.W. (2011). Breast cancer-induced bone remodeling, skeletal pain, and sprouting of sensory nerve fibers. J. Pain.

[95] Lane N.E., Schnitzer T.J., Birbara C.A., Mokhtarani M., Shelton D.L., Smith M.D., Brown M.T. (2010). Tanezumab for the treatment of pain from osteoarthritis of the knee. N. Engl. J. Med..

[96] Schnitzer T.J., Ekman E.F., Spierings E.L., Greenberg H.S., Smith M.D., Brown M.T., West C.R., Verburg K.M. (2015). Efficacy and safety of tanezumab monotherapy or combined with non-steroidal anti-inflammatory drugs in the treatment of knee or hip osteoarthritis pain. Ann. Rheum. Dis..

[97] Katz N., Borenstein D.G., Birbara C., Bramson C., Nemeth M.A., Smith M.D., Brown M.T. (2011). Efficacy and safety of tanezumab in the treatment of chronic low back pain. Pain.

[98] Bramson C., Herrmann D.N., Carey W., Keller D., Brown M.T., West C.R., Verburg K.M., Dyck P.J. (2015). Exploring the role of tanezumab as a novel treatment for the relief of neuropathic pain. Pain Med..

[99] Sopata M., Katz N., Carey W., Smith M.D., Keller D., Verburg K.M., West C.R., Wolfram G., Brown M.T. (2015). Efficacy and safety of tanezumab in the treatment of pain from bone metastases. Pain.

[100] Bunnett N.W. (2006). Protease-activated receptors: How proteases signal to cells to cause inflammation and pain. Semin. Thromb. Hemost..

[101] Vergnolle N., Bunnett N.W., Sharkey K.A., Brussee V., Compton S.J., Grady E.F., Cirino G., Gerard N., Basbaum A.I., Andrade-Gordon P. (2001). Proteinase-activated receptor-2 and hyperalgesia: A novel pain pathway. Nat. Med..

[102] Mrozkova P., Palecek J., Spicarova D. (2016). The role of protease-activated receptor type 2 in nociceptive signaling and pain. Physiol. Res..

[103] Reed D.E., Barajas-Lopez C., Cottrell G., Velazquez-Rocha S., Dery O., Grady E.F., Bunnett N.W., Vanner S.J. (2003). Mast cell tryptase and proteinase-activated receptor 2 induce hyperexcitability of guinea-pig submucosal neurons. J. Physiol..

[104] Molino M., Barnathan E.S., Numerof R., Clark J., Dreyer M., Cumashi A., Hoxie J.A., Schechter N., Woolkalis M., Brass L.F. (1997). Interactions of mast cell tryptase with thrombin receptors and par-2. J. Biol. Chem..

[105] Nystedt S., Emilsson K., Wahlestedt C., Sundelin J. (1994). Molecular cloning of a potential proteinase activated receptor. Proc. Natl. Acad. Sci. USA.

[106] Kleij H.P., Bienenstock J. (2005). Significance of conversation between mast cells and nerves. Allergy Asthma Clin. Immunol..

[107] Barbara G., Stanghellini V., De Giorgio R., Cremon C., Cottrell G.S., Santini D., Pasquinelli G., Morselli-Labate A.M., Grady E.F., Bunnett N.W. (2004). Activated mast cells in proximity to colonic nerves correlate with abdominal pain in irritable bowel syndrome. Gastroenterology.

[108] Demir I.E., Schorn S., Schremmer-Danninger E., Wang K., Kehl T., Giese N.A., Algul H., Friess H., Ceyhan G.O. (2013). Perineural mast cells are specifically enriched in pancreatic neuritis and neuropathic pain in pancreatic cancer and chronic pancreatitis. PLoS ONE.

[109] Lam D.K., Schmidt B.L. (2010). Serine proteases and protease-activated receptor 2-dependent allodynia: A novel cancer pain pathway. Pain.

[110] Mantyh P.W. (2014). The neurobiology of skeletal pain. Eur. J. Neurosci..

[111] Hong D., Byers M.R., Oswald R.J. (1993). Dexamethasone treatment reduces sensory neuropeptides and nerve sprouting reactions in injured teeth. Pain.

[112] Ghilardi J.R., Freeman K.T., Jimenez-Andrade J.M., Coughlin K.A., Kaczmarska M.J., Castaneda-Corral G., Bloom A.P., Kuskowski M.A., Mantyh P.W. (2012). Neuroplasticity of sensory and sympathetic nerve fibers in a mouse model of a painful arthritic joint. Arthritis Rheum.

[113] Chartier S.R., Thompson M.L., Longo G., Fealk M.N., Majuta L.A., Mantyh P.W. (2014). Exuberant sprouting of sensory and sympathetic nerve fibers in nonhealed bone fractures and the generation and maintenance of chronic skeletal pain. Pain.

[114] Riesco N., Cernuda-Morollon E., Pascual J. (2017). Neuropeptides as a marker for chronic headache. Curr. Pain Headache Rep..

[115] Schou W.S., Ashina S., Amin F.M., Goadsby P.J., Ashina M. (2017). Calcitonin gene-related peptide and pain: A systematic review. J. Headache Pain.

[116] Liu S., Liu Y.P., Yue D.M., Liu G.J. (2014). Protease-activated receptor 2 in dorsal root ganglion contributes to peripheral sensitization of bone cancer pain. Eur. J. Pain.

[117] Leporini C., Ammendola M., Marech I., Sammarco G., Sacco R., Gadaleta C.D., Oakley C., Russo E., De Sarro G., Ranieri G. (2015). Targeting mast cells in gastric cancer with special reference to bone metastases. World J. Gastroenterol..

[118] Ammendola M., Marech I., Sammarco G., Zuccala V., Luposella M., Zizzo N., Patruno R., Crovace A., Ruggieri E., Zito A.F. (2015). Infiltrating mast cells correlate with angiogenesis in bone metastases from gastric cancer patients. Int. J. Mol. Sci..

[119] Tondevold E., Eriksen J., Jansen E. (1979). Observations on long bone medullary pressure in relation to mean arterial blood pressure in the anaesthetized dog. Acta Orthop. Scand..

[120] Hu J., Van Valckenborgh E., Menu E., De Bruyne E., Vanderkerken K. (2012). Understanding the hypoxic niche of multiple myeloma: Therapeutic implications and contributions of mouse models. Dis. Models Mech..

[121] Wang Y., Wan C., Deng L., Liu X., Cao X., Gilbert S.R., Bouxsein M.L., Faugere M.C., Guldberg R.E., Gerstenfeld L.C. (2007). The hypoxia-inducible factor alpha pathway couples angiogenesis to osteogenesis during skeletal development. J. Clin. Investig..

[122] Wan C., Gilbert S.R., Wang Y., Cao X., Shen X., Ramaswamy G., Jacobsen K.A., Alaql Z.S., Eberhardt A.W., Gerstenfeld L.C. (2008). Activation of the hypoxia-inducible factor-1alpha pathway accelerates bone regeneration. Proc. Natl. Acad. Sci. USA.

[123] Shomento S.H., Wan C., Cao X., Faugere M.C., Bouxsein M.L., Clemens T.L., Riddle R.C. (2010). Hypoxia-inducible factors 1alpha and 2alpha exert both distinct and overlapping functions in long bone development. J. Cell. Biochem..

[124] Knowles H.J., Athanasou N.A. (2009). Acute hypoxia and osteoclast activity: A balance between enhanced resorption and increased apoptosis. J. Pathol..

[125] Rankin E.B., Giaccia A.J., Schipani E. (2011). A central role for hypoxic signaling in cartilage, bone, and hematopoiesis. Curr. Osteoporos. Rep..

[126] Vander Heiden M.G., Cantley L.C., Thompson C.B. (2009). Understanding the warburg effect: The metabolic requirements of cell proliferation. Science.

[127] Peppicelli S., Bianchini F., Toti A., Laurenzana A., Fibbi G., Calorini L. (2015). Extracellular acidity strengthens mesenchymal stem cells to promote melanoma progression. Cell Cycle.

[128] Di Pompo G., Lemma S., Canti L., Rucci N., Ponzetti M., Errani C., Donati D.M., Russell S., Gillies R., Chano T. (2017). Intratumoral acidosis fosters cancer-induced bone pain through the activation of the mesenchymal tumor-associated stroma in bone metastasis from breast carcinoma. Oncotarget.

[129] Montazeri A. (2009). Quality of life data as prognostic indicators of survival in cancer patients: An overview of the literature from 1982 to 2008. Health Qual. Life Outcomes.

[130] Halabi S., Vogelzang N.J., Kornblith A.B., Ou S.S., Kantoff P.W., Dawson N.A., Small E.J. (2008). Pain predicts overall survival in men with metastatic castration-refractory prostate cancer. J. Clin. Oncol..

[131] Koizumi M., Yoshimoto M., Kasumi F., Iwase T., Ogata E. (2010). Post-operative breast cancer patients diagnosed with skeletal metastasis without bone pain had fewer skeletal-related events and deaths than those with bone pain. BMC Cancer.

[132] Fizazi K., Massard C., Smith M., Rader M., Brown J., Milecki P., Shore N., Oudard S., Karsh L., Carducci M. (2015). Bone-related parameters are the main prognostic factors for overall survival in men with bone metastases from castration-resistant prostate cancer. Eur. Urol..

[133] Niikura N., Liu J., Hayashi N., Palla S.L., Tokuda Y., Hortobagyi G.N., Ueno N.T., Theriault R.L. (2011). Treatment outcome and prognostic factors for patients with bone-only metastases of breast cancer: A single-institution retrospective analysis. Oncologist.

[134] Parker C., Nilsson S., Heinrich D., Helle S.I., O’Sullivan J.M., Fossa S.D., Chodacki A., Wiechno P., Logue J., Seke M. (2013). Alpha emitter radium-223 and survival in metastatic prostate cancer. N. Engl. J. Med..

[135] Boilly B., Faulkner S., Jobling P., Hondermarck H. (2017). Nerve dependence: From regeneration to cancer. Cancer Cell.

[136] Magnon C., Hall S.J., Lin J., Xue X., Gerber L., Freedland S.J., Frenette P.S. (2013). Autonomic nerve development contributes to prostate cancer progression. Science.

[137] Hayakawa Y., Sakitani K., Konishi M., Asfaha S., Niikura R., Tomita H., Renz B.W., Tailor Y., Macchini M., Middelhoff M. (2017). Nerve growth factor promotes gastric tumorigenesis through aberrant cholinergic signaling. Cancer Cell.

[138] Zhao C.M., Hayakawa Y., Kodama Y., Muthupalani S., Westphalen C.B., Andersen G.T., Flatberg A., Johannessen H., Friedman R.A., Renz B.W. (2014). Denervation suppresses gastric tumorigenesis. Sci. Transl. Med..

[139] Saloman J.L., Albers K.M., Li D., Hartman D.J., Crawford H.C., Muha E.A., Rhim A.D., Davis B.M. (2016). Ablation of sensory neurons in a genetic model of pancreatic ductal adenocarcinoma slows initiation and progression of cancer. Proc. Natl. Acad. Sci. USA.

[140] Bao Y., Hou W., Yang L., Kong X., Du M., Zheng H., Gao Y., Hua B. (2015). Protease-activated receptor 2 antagonist potentiates analgesic effects of systemic morphine in a rat model of bone cancer pain. Reg. Anesth. Pain Med..

